# Comparative Evaluation of the Efficacy of Light-Cured Calcium Hydroxide and a Fourth-Generation Calcium Silicate Cement (TheraCal LC) as Indirect Pulp Capping Materials in Patients With Deep Carious Lesions: A Randomized Parallel-Group Clinical Trial

**DOI:** 10.7759/cureus.28882

**Published:** 2022-09-07

**Authors:** Joyeeta Mahapatra, Pradnya Nikhade, Aditya Patel, Prachi Taori, Kajol Relan

**Affiliations:** 1 Department of Conservative Dentistry and Endodontics, Sharad Pawar Dental College, Datta Meghe Institute of Medical Sciences (Deemed to be University), Wardha, IND

**Keywords:** calcific barrier, calcium silicate cement, calcium hydroxide, vital pulp therapy, : indirect pulp capping

## Abstract

Aim

The current study aims to evaluate and compare the efficacy of light-cured calcium hydroxide and a fourth-generation calcium silicate cement (TheraCal LC®) as indirect pulp capping (IPC) materials in patients with deep carious lesions.

Materials and methods

A total of 28 patients were randomly divided into two groups (n=14). Group A was managed by light-cured calcium hydroxide, while group B was treated with TheraCal LC (a fourth-generation calcium silicate cement). Clinical examination was conducted to check for postoperative pain, tenderness, and neural sensibility, and radiographical examination was conducted to check for periodontal ligament space widening, presence of calcific barrier, and periapical radiolucency at patient recall of 21 days, three months, and six months. Primary and secondary outcome variables were based on clinical and radiographical success rates noted at six months’ follow-up.

Results

Success rate for light-cured calcium hydroxide group at follow-up came out to be 0% at 21 days, 85.71% at three months, and 92.85% at six months. The success rate for TheraCal LC group came out to be 0% at 21 days, 92.85% at three months, and 100% at six months. The overall success rate for IPC procedure was 89.28% at three months’ follow-up and 96.42% at six months’ follow-up for both groups. The difference was statistically non-significant at the end of three and six months’ follow-up.

Conclusion

Within the limitations of our study, it was concluded that TheraCal LC can be used alternatively with light-cured calcium hydroxide in IPC, with a predictability of similar success outcome in patients with deep carious lesions.

## Introduction

Indirect pulp capping (IPC) is a treatment modality that aids in sustaining pulpal vitality by facilitating healing/repair [1] of traumatized pulp. Such procedures have been quite challenging for the clinician because it involves removal of caries approximating the pulpal tissue. Care should be taken to not expose the pulp chamber during the excavation process. IPC is the treatment of choice for deeply decayed teeth.

IPC agent is placed at the affected site with an objective to form "dentin‑like matrix" (reactionary/reparative dentin). This helps in repairing the "dentin‑pulp complex." There is stimulation of dentinal sclerosis, which results in formation of reparative dentin [2]. Also, there is arresting of demineralization of dentin by the carious process [3]. This leads to the preservation of pulp vitality. There has been promotion of a variety of materials as pulp capping agents for managing vital teeth with deep carious lesions.

Calcium hydroxide (CH) is taken as the gold standard among all the pulp capping agents that are used. Its use was first put forward by Zander [4]. There is formation of "reparative dentin," which takes place by cellular differentiation and extracellular matrix secretion. Subsequently, there is mineralization. Following this cascade of mechanism, a lot of drawbacks are seen. For example, there is an appearance of "tunnel defects" by the slow disintegrating "newly formed dentin." This phenomenon can be observed with CH when a long follow-up is taken [5]. CH was found to be successful clinically by 97.6% after 16 months of follow-up [6] and by 13% after 10 years of follow-up [7]. This paved way for other IPC materials such as calcium silicate-based materials (mineral trioxide aggregate, Biodentine®, TheraCal LC®, etc.).

On the basis of the chemical properties and composition of calcium silicate cement, TheraCal LC is classified as a fourth-generation calcium silicate-based material [8]. It is a single syringe system available in paste consistency. Depending on the case, it is used either as a pulp capping agent or as a protective liner in direct restorations [9]. According to ISO 9917-2017 part 2 clause 4.1, "TheraCal LC is a class 2 cement material in which the setting reaction of the polymerizable component is light activated" [9]. It has high calcium-releasing ability and can be cured to a depth of 1.7 mm to prevent dissolution [8]. According to Chaudhari et al. [10] in their in vitro study, TheraCal LC has been found as a better Ca-ion releasing material than light-cured CH [10].

Only a few clinical trials have been conducted so far, determining the clinical efficacy and successful outcome of light-cured CH or TheraCal LC as IPC agents in permanent teeth with deep caries. Hence, there is a requirement for more studies on these two materials. The objective of our study was to evaluate clinically postoperative pain, tenderness, and neural sensibility, and to check radiographically periodontal ligament (PDL) space widening, periapical radiolucency, and the presence of calcified barrier using intraoral periapical radiograph (IOPA). This evaluation was carried out at follow-up interval of 21 days, three months, and six months after IPC with light-cured CH and TheraCal LC in permanent teeth with deep carious lesions. The other objective of the study is the comparison of the efficacy of these two materials as IPC agents.

This article was previously presented as a meeting abstract at the 12th IFEA WEC 202One on August 8, 2021.

## Materials and methods

Study design

**Figure 1:**
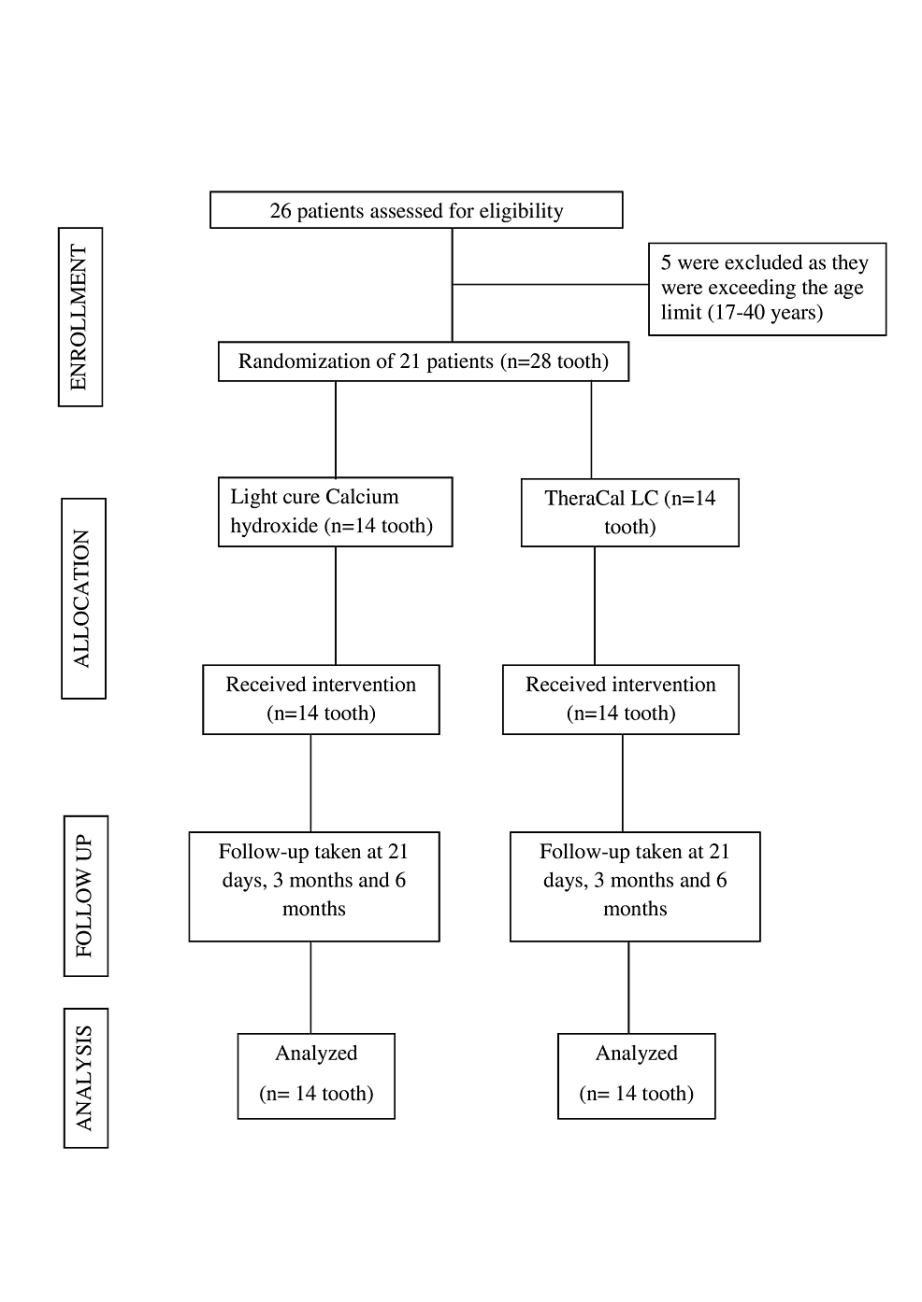
Consolidated Standards of Reporting Trials flowchart of participants throughout the trial

This randomized, parallel-group trial was carried out in the Department of Conservative Dentistry and Endodontics, Sharad Pawar Dental College and Hospital, Sawangi (Meghe), Wardha, India. It got its approval from the Institutional Ethics Committee of Datta Meghe Institute of Medical Sciences (Deemed to be University) (Ref No.- DMIMS(DU)/IEC/Dec-2019/8562) and was registered at Clinical Trial Registry-India as CTRI/2020/03/023808. Study participants were recruited from the patient group that reported at the Outpatient Department, where they were explained the significance of IPC. Before their enrolment in the clinical trial, participants were explained the potential risks and the expected benefits of treatment procedure. Informed consent was taken prior to the commencement of the treatment procedure. Confidentiality related to the participants was maintained. Sequence generation for the usage of the two materials was done by computer-generated randomization. Centralized allocation of the materials was done for the study. The reporting of the trial has been done according to CONSORT 2010 statement: updated guidelines for reporting parallel group randomized trials (Figure 1).

Inclusion criteria

Participants of the age group of 17-40 years, who consented, got selected for the study. Only those with matured permanent tooth with closed apex, having caries penetrating in dentin in more than 1/2 or 3/4th the thickness of the dentinal bulk, as observed on IOPA, and diagnosed as "reversible pulpitis" were included in the study [11]. The tooth samples should give a positive response to cold test.

Exclusion criteria

This study excluded deciduous teeth, teeth having pulpal necrosis, or teeth with the diagnosis of irreversible pulpitis with/without periapical lesion as seen on IOPA, those with periodontitis, those with teeth showing internal or external resorption, those having calcified canals, cracked teeth, or teeth that failed to attain hemostasis after inadvertent pinpoint pulpal exposure, immunocompromised or pregnant patients, and patients taking antibiotic/analgesic within one week before the commencement of the treatment [11].

Sample size

On the basis of the results of former studies and depending on the percentage of success of direct pulp capping with mineral trioxide aggregate as 80% [12] versus CH as 33.3% [10] using α=5% with a power of 80%, the sample size was calculated using the following formula:

n = [Zα/2 +Z1-β]2 [p1 (1-p1) + p2(1-p2)] / (p1-p2) 2

The sample size was 14 in each group. Therefore, a total of 28 samples were required.

Treatment protocol

A detailed case history was taken, and the patients were thoroughly examined both clinically and radiologically. Local anesthesia (2% lignocaine HCl with epinephrine) was administered to prevent unpleasantness of sensitivity during caries removal. There was reduction of postoperative pain or sensitivity as well. The sample tooth was isolated with a rubber dam. Using a slow-speed round bur, the superficial caries layer was removed. Disinfection of cavity was done with 2% chlorhexidine (Safe Plus, Gujarat, India) followed by irrigation with saline. The rest of the soft caries present in the deep layers was removed carefully with a spoon excavator. Disinfection of the rest of cavity was done by irrigation with 3% NaOCl (Parcan, Septodont, Maharashtra, India) with the help of a syringe. The cavity was rinsed with saline to get rid of the excess NaOCl. The cavity was dried using cotton pellets [11]. Pulp capping material was chosen and placed in patients as per the sequence generated in randomization. They were placed as per their manufacturer's instructions.

**Figure 2:**
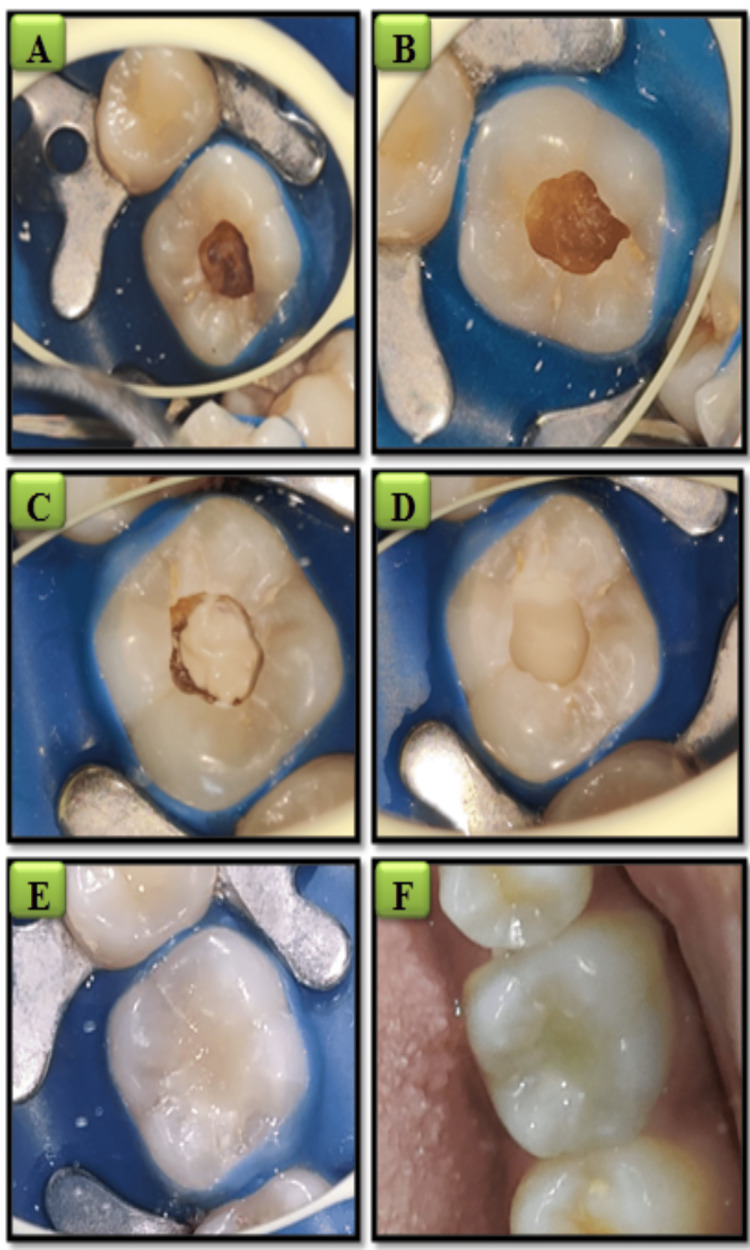
Clinical procedure for indirect pulp capping. (A) Rubber dam isolation in relation to 36. (B) Caries excavation done. (C) Pulp capping agent placed on the exposed site. (D) Glass ionomer cement base given. (E) Final restoration done with resin composite. (F) Postoperative clinical photograph after finishing and polishing.

In the light-cured CH (Prevest Cal LC DenPro, Brussels, Belgium) group, CH was placed at the exposed area followed by its curing. Similarly, in the TheraCal LC (Bisco Inc., Schaumburg, IL, USA) group, the agent was placed on the affected site and cured. Type II glass ionomer cement (GIC; GC, Fuji, Japan) was used as a base. Following this, a direct composite restoration (Spectrum microhybrid composite, Dentsply, New York, USA) was undertaken. Finishing of the final restoration was done to attain a smoother surface to decrease plaque aggregation and to prevent discoloration of the restorative material [13] (Figure 2). After finishing the procedure, an IOPA of the tooth was recorded. This was kept as a baseline for the future follow-up [11].

Follow-up and outcome measures

The tooth was examined clinically to evaluate postoperative pain, tenderness, and neural sensibility, and radiographically to assess PDL space widening, presence of calcified barrier, and periapical radiolucency at a follow-up interval of 21 days, three months, and six months. On the basis of outcomes of evaluation of primary and secondary variables, at follow-up intervals, the clinical and radiographical success rate was determined. A successful primary outcome was indicated by the presence of calcified barrier at the restored site. A successful secondary outcome was defined by the absence of postoperative pain, tenderness on percussion, and positive response to neural sensibility tests, with absence of any radiographical signs of PDL space widening and lack of periapical radiolucency. The experience of pain by participants was scored using visual analogue scale (VAS) before and after the procedure [11].

Patients were recalled for follow-up at the intervals of 21 days, three months, and six months postoperatively. During this session, they were assessed clinically for postoperative pain using VAS. Assessment of tenderness was done by vertical percussion of the restored tooth. Endo-Ice (Coltene-Whaledent, Cuyahoga Falls, OH, USA) was used for cold test in order to evaluate neural sensibility.

**Figure 3:**
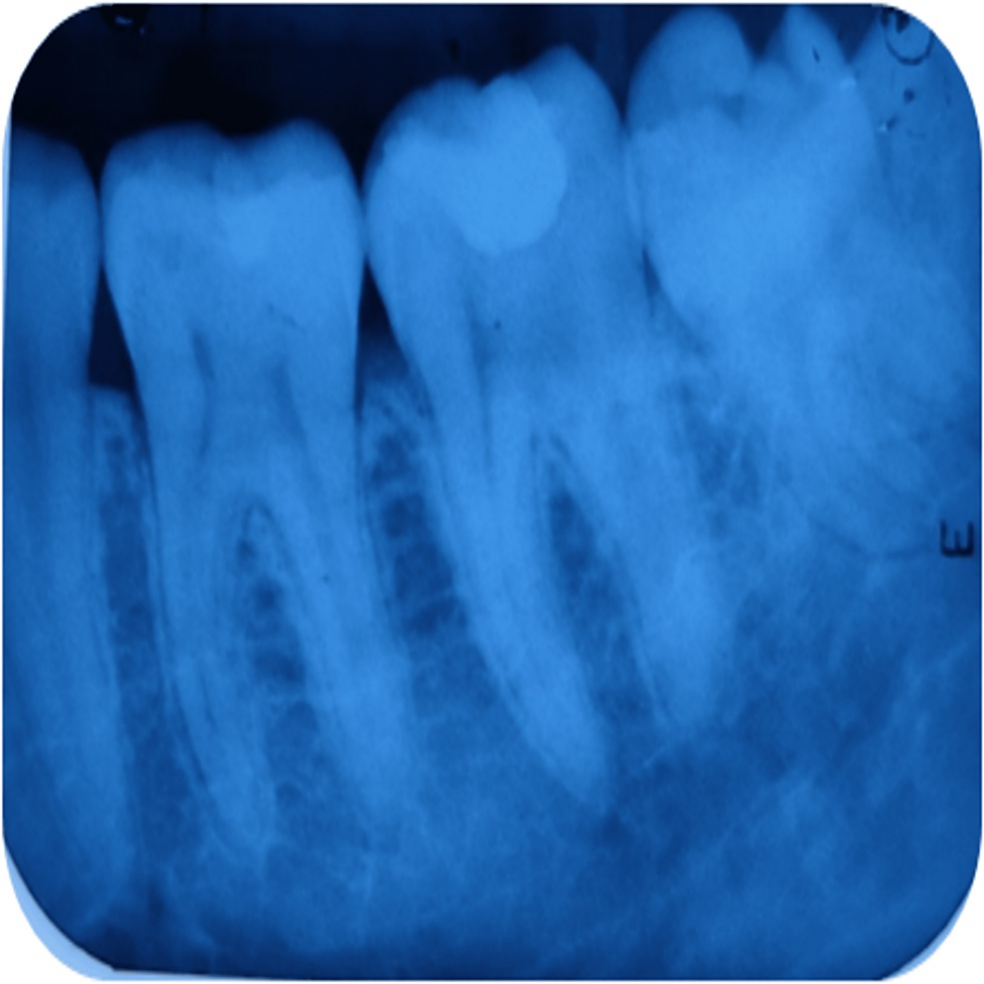
Radiographical assessment of light-cured calcium hydroxide using intraoral periapical radiograph at 21 days recall in relation to 37.

**Figure 4:**
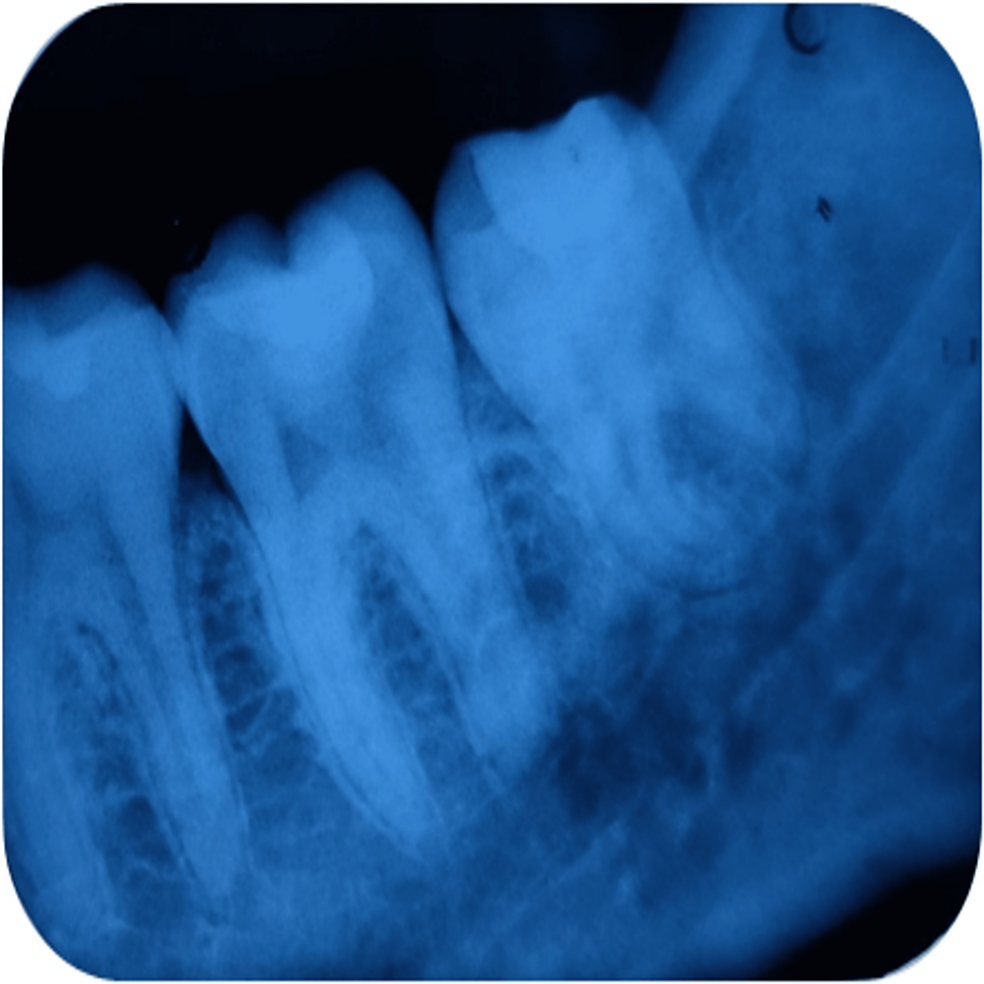
Radiographical assessment of light-cured calcium hydroxide using intraoral periapical radiograph at three months' recall in relation to 37.

**Figure 5:**
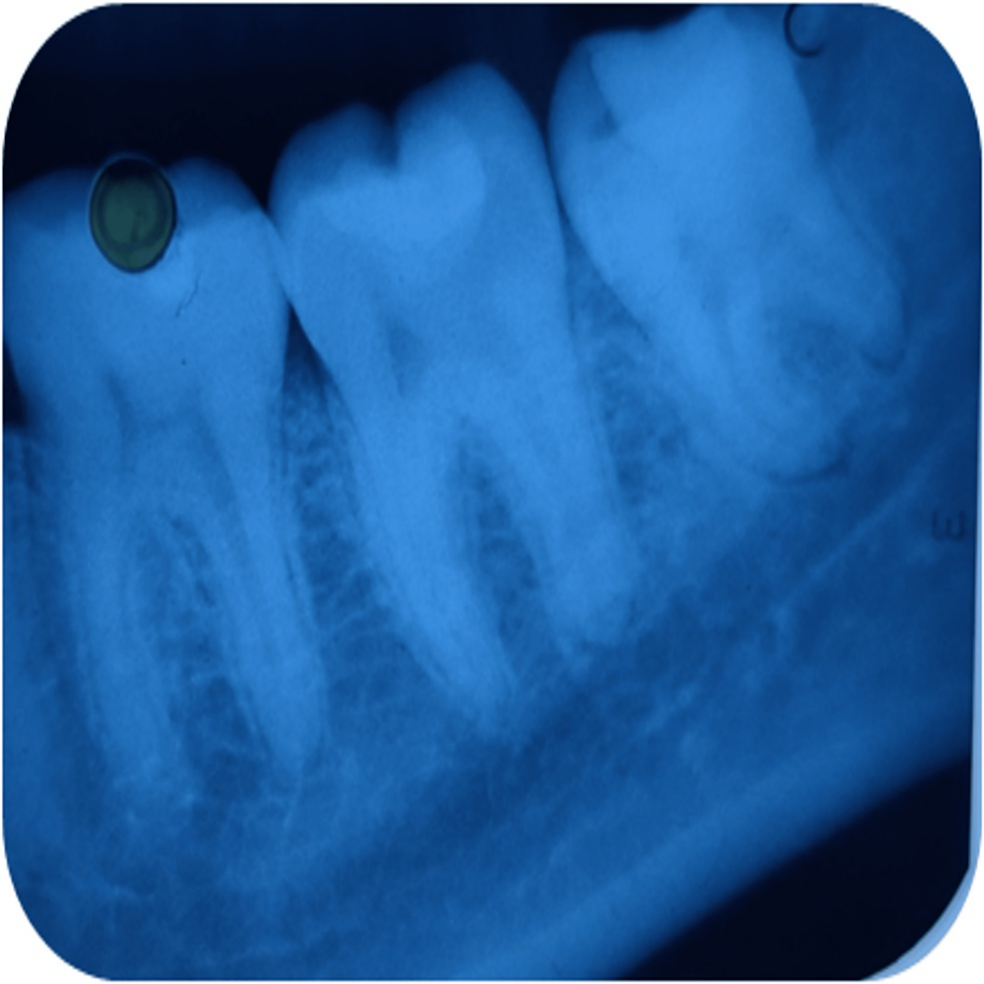
Radiographical assessment of light-cured calcium hydroxide using intraoral periapical radiograph at six months' recall in relation to 37.

**Figure 6:**
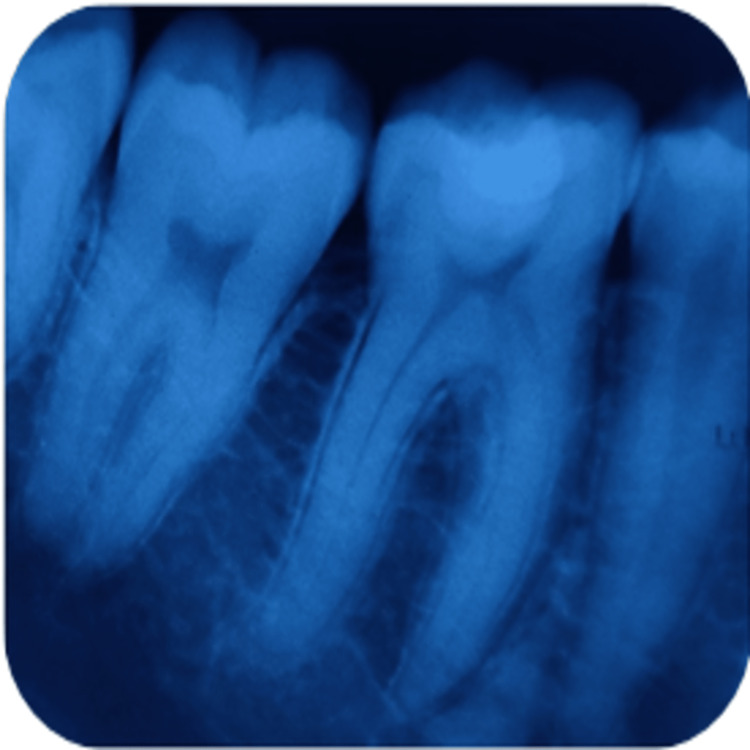
Radiographical assessment of TheraCal LC using intraoral periapical radiograph at 21 days recall in relation to 46.

**Figure 7:**
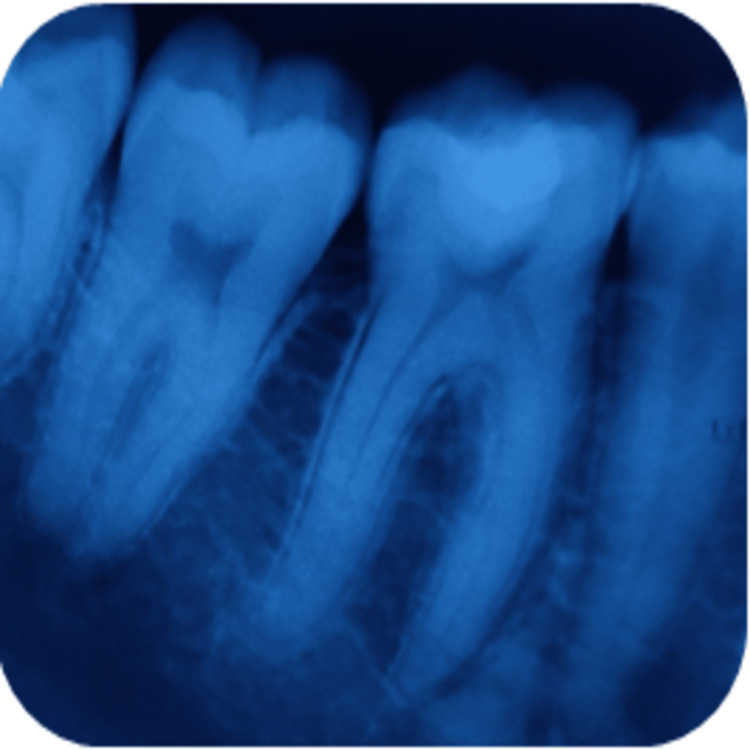
Radiographical assessment of TheraCal LC using intraoral periapical radiograph at three months' recall in relation to 46.

**Figure 8:**
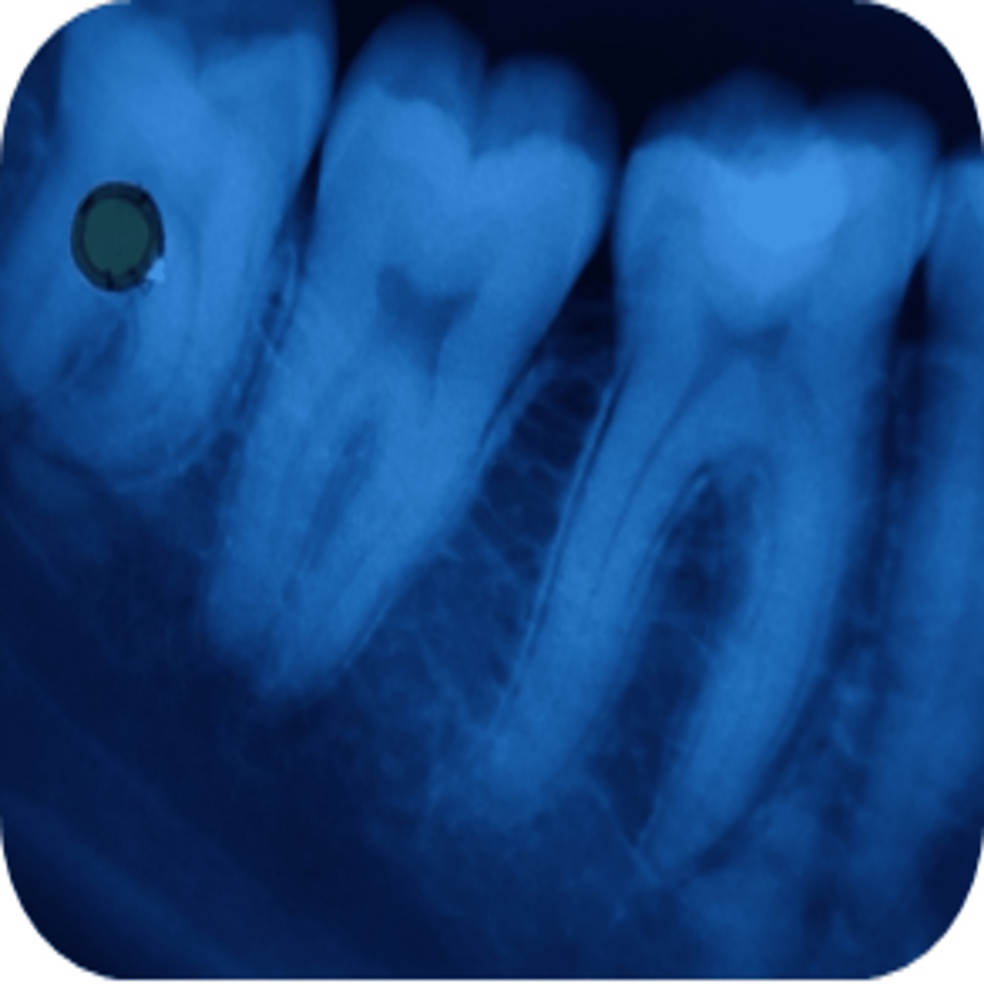
Radiographical assessment of TheraCal LC using intraoral periapical radiograph at six months' recall in relation to 46.

Also, for assessing the tooth radiographically, periapical radiograph was recorded during these follow-up intervals. Evaluated parameters were presence/absence of PDL space widening, presence/ absence of periapical radiolucency, and the presence/absence of calcified barrier formation below the restoration. Radiographical assessment of group A at different time periods of follow-up is depicted in Figures 3-5 and that of group B is depicted in Figures 6-8.

 

Statistical analysis

Descriptive and analytical statistics had been done. The normality of data distribution had been analyzed using the Shapiro-Wilk test (Statistics Version 20, IBM Corp., Armonk, NY). As data followed normal distribution, parametric tests were utilized for analyzing the data. Fisher's exact test was applied for dichotomous variables such as tenderness on percussion, neural sensibility, PDL space widening, presence of calcified barrier, periapical radiolucency, and success rate.

The one-way analysis of variance (ANOVA) test was utilized for the parameter of pain for checking the mean differences between the groups at different time period, and "post hoc Tukey’s HSD (honestly significant difference) test was used to compare the difference between two groups. The level of significance was set at less than 0.05 using two-tailed analysis. Statistical analysis was conducted using SPSS (Statistical Package for Social Sciences) Version 24.0 (IBM Corp.).

## Results

A total of 26 patients were screened for the study, of which five were excluded because their age did not meet the age limit criteria. Therefore, only 21 participants were kept in the trial. They gave us the required sample size of 28 teeth. They were randomly allocated to the light cure CH group (group A) and the TheraCal LC group (group B), i.e., 14 teeth samples in each group. Among the teeth types, 10.7% comprised anterior teeth, 17.85% comprised premolars, 28.57% comprised first molars, 25% comprised second molars, and 17.88% comprised third molars.

At the end of three months, 12 out of 14 teeth and 13 out of 14 teeth exhibited calcific barrier formation in the light-cured CH group and the TheraCal LC group, respectively. By the sixth month, 13 out of 14 teeth and all 14 teeth displayed calcific barrier in the light-cured CH group and the TheraCal LC group, respectively (Table 1). Only one case showed failure in the light-cured CH group. The tooth had mild pain by the third month (Table 2). And at the end of 6 months, it developed tenderness on percussion, was non-responsive to neural sensibility testing, and exhibited PDL space widening in periapical radiograph (Tables 1, 2). In this case, root canal treatment was performed.

**Table 1:** Intergroup comparison for radiographical parameter T1, 21 days; T2, 3 months; T3, 6 months

Group	T1	T2	T3
Presence of calcified barrier			
A	0	12 (85.71%)	13 (92.45%)
B	0	13 (92.45%)	14 (100%)
Periodontal ligament space widening			
A	0	0	1 (7.14%)
B	0	0	0
Presence of periapical radiolucency			
A	0	0	0
B	0	0	0

**Table 2:** Intergroup comparison for clinical parameter T1, 21 days; T2, 3 months; T3, 6 months

Group	T1	T2	T3
Pain using visual analogue scale			
A	0	1 (7.14%)	1 (7.14%)
B	0	0	0
Tenderness on percussion			
A	0	0	1 (7.14%)
B	0	0	0
Neural sensibility test (cold test)			
A	0	0	1 (7.14%)
B	0	0	0

The success rate for the light-cured CH group (Cal LC) at follow-up was found to be 0% at 21 days, 85.71% at three months, and 92.85% at six months. The success rate for the TheraCal LC group was found to be 0% at 21 days, 92.85% at three months, and 100% at six months (Table 3).

**Table 3:** Intergroup comparison for overall success rate T1, 21 days; T2, 3 months; T3, 6 months

Group	T1	T2	T3
A	0	12 (85.71%)	13 (92.45%)
B	0	13 (92.45%)	14 (100%)
Total	0	25/28 (89.28%)	27 (96.42%)
p-Value	1		

The overall success rate for IPC procedure was 89.28% at three’ follow-up months and 96.42% at six months’ follow-up for both groups. The difference was statistically non-significant at the end of three and six months’ follow-up.

## Discussion

Treatment of reversible pulp injury is the main aim of indirect IPC. After caries removal, the thin layer of dentin remaining between the prepared cavity and the pulp chamber is lined with a protective covering to prevent any further injury to the pulp and to facilitate its healing and repair. The type of treatment strategy has a key function in determining the success of vital pulp therapy. It includes the operative procedure undertaken to remove the pulpal irritant, controlling infection, and placement of a protective barrier for isolation of pulp/dentin from further injuries [1]. Also, the material used in the treatment modality plays an important role in determining the biological process that would stimulate a specific dentin response that would lead to calcified barrier formation. In our case, the calcium silicate cement (TheraCal LC) used encourages dentine bridge formation by secreting transforming growth factor (TGF)-b1. There is no inflammatory pulp response involved in the procedure [14,15]. On the other hand, light-cured CH (Ca(OH)2) releases hydroxyl (OH-) and calcium (Ca+) ions on dissolution. The alkalinity of this pulp protective agent has a pH of 9-11 that induces forming of secondary/reparative dentin [10]. Restoration of the cavity to achieve adequate sealing in order to prevent microleakage is also a key step. A base of light-cured GIC is given followed by composite restoration. For final restoration, resin composite is used. It provides good bond strength and is resistant to solubility. Also, there is a decreased incidence of secondary carious lesions in comparison to GIC [16]. Vital pulp therapy preserves the pulp vitality and the structural and functional integrity of the pulp-dentin complex [1]. We cannot rely on any objective means during clinical evaluation of the extension of pulpal inflammation. Identification of pulpal condition depends on the patient's description of symptoms, results of neural sensibility test, and radiographical examination [17,18].

Ca(OH)2 was introduced as a "remineralizing agent" for vital pulp therapy in 1930. It aids in the production of reparative dentin in the treated site by releasing Ca+ and OH- ions. The calcium ions form a gradient that stimulate the aggregation of undifferentiated cells of the pulp. Along with this, there is activation of stem cells. An alkaline condition is created. This type of environment is bacteriostatic and triggers the action of alkaline phosphatase and bone morphogenic protein‑2 (BMP‑2). This favors the production of calcific nodules. Calcium ions thus help differentiate and mineralize pulp cells [19]. Aggregation of pulp cells is directly proportional to the release of calcium ions [20,21]. Additionally, Ca+ modulates osteopontin and BMP-2 level. This has a role in pulp calcification [22]. The formation of calcified barrier or dentin bridge requires pyrophosphatase whose activity is facilitated by calcium ion [23]. As per a research study by Chaudhari et al., the quantity of calcium ion released from TheraCal is >4 mmol/L. This amount is sufficient enough to trigger the activity of dental pulp cells and odontoblasts [10]. This study also stated that TheraCal LC is a higher calcium ion releasing agent than Cal LC [10].

In our study, it was found that TheraCal LC has a slightly better outcome as compared to light cure CH group, with a success rate of 100% and 92.85%, respectively, at the end of six months’ follow-up. The difference was statistically non-significant at the end of three and three months’ follow-up.

A few limitations were present in the study. The follow-up carried out in the study was up to six months. This was a short period to determine the success rate of IPC [5]. Also, no 3D imaging technique, such as cone beam computed tomography, was used in the study for the visualization of the calcified barrier.

## Conclusions

The objective of restoring deep carious lesions is the preservation and maintenance of pulp vitality after it has been subjected to events such as trauma, carious process, or mechanical insults due to iatrogenic errors. IPC is a technique that aids in preserving a vital pulp and restoring it to its healthy state. Within the limitations of the study, as per our observation, it was concluded that TheraCal LC can be used alternatively with light-cured CH in IPC, with a predictability of similar successful outcome in patients with deep carious lesions. In the quest of search for better materials for IPC, continuous developments in the field of conservative dentistry had led to invention of newer pulp capping agents. These agents play a significant role in maintaining vitality of pulp tissue, thus preventing more invasive intervention for the tooth.

## References

[1] Tziafas D, Smith AJ, Lesot H (2000). Designing new treatment strategies in vital pulp therapy. J Dent.

[2] Bogen G, Kim JS, Bakland LK (2008). Direct pulp capping with mineral trioxide aggregate: an observational study. J Am Dent Assoc.

[3] Brizuela C, Ormeño A, Cabrera C, Cabezas R, Silva CI, Ramírez V, Mercade M (2017). Direct pulp capping with calcium hydroxide, mineral trioxide aggregate, and biodentine in permanent young teeth with caries: a randomized clinical trial. J Endod.

[4] Zander HA (1939). Reaction of the pulp to calcium hydroxide. J Dent Res.

[5] Hashem D, Mannocci F, Patel S, Manoharan A, Brown JE, Watson TF, Banerjee A (2015). Clinical and radiographic assessment of the efficacy of calcium silicate indirect pulp capping: a randomized controlled clinical trial. J Dent Res.

[6] Armstrong WP, Hoffman S (1962). Pulp-cap study. Oral Surg Oral Med Oral Pathol Oral Radiol.

[7] Barthel CR, Rosenkranz B, Leuenberg A, Roulet JF (2000). Pulp capping of carious exposures: treatment outcome after 5 and 10 years: a retrospective study. J Endod.

[8] Dutta A, Saunders WP (2014). Calcium silicate materials in endodontics. Dent Update.

[9] Arandi NZ, Rabi T (2018). TheraCal LC: from biochemical and bioactive properties to clinical applications. Int J Dent.

[10] Chaudhari W, Jain R, Jadhav S, Hegde V, Dixit M (2016). Calcium ion release from four different light-cured calcium hydroxide cements. Endodontology.

[11] Mahapatra J, Pradnya P, Sukhtankar S (2020). Comparative evaluation of the efficacy of light cure calcium hydroxide and theracal lc as indirect pulp capping materials in patients with deep carious lesion - an interventional study. Eur J Mol Clin Med.

[12] Mente J, Hufnagel S, Leo M (2014). Treatment outcome of mineral trioxide aggregate or calcium hydroxide direct pulp capping: long-term results. J Endod.

[13] Sathe S, Karva S, Borle A, Dhamande M, Jaiswal T, Nimonkar S (2019). "Comparative evaluation of the effect of three polishing agents on staining characteristics of provisional restorative material:" an in vitro study. J Int Soc Prev Community Dent.

[14] Laurent P, Camps J, About I (2012). Biodentine(TM) induces TGF-β1 release from human pulp cells and early dental pulp mineralization. Int Endod J.

[15] Nowicka A, Lipski M, Parafiniuk M (2013). Response of human dental pulp capped with biodentine and mineral trioxide aggregate. J Endod.

[16] Rathi NV, Chandak MG, Mude GA (2018). Comparative evaluation of dentinal caries in restored cavity prepared by galvanic and sintered burs. Contemp Clin Dent.

[17] Bjørndal L (2002). Dentin and pulp reactions to caries and operative treatment: biological variables affecting treatment outcome. Endod Topics.

[18] Pitt Ford TR, Patel S (2004). Technical equipment for assessment of dental pulp status. Endod Topics.

[19] Schröder U (1985). Effects of calcium hydroxide-containing pulp-capping agents on pulp cell migration, proliferation, and differentiation. J Dent Res.

[20] Takita T, Hayashi M, Takeichi O, Ogiso B, Suzuki N, Otsuka K, Ito K (2006). Effect of mineral trioxide aggregate on proliferation of cultured human dental pulp cells. Int Endod J.

[21] Lopez-Cazaux S, Bluteau G, Magne D, Lieubeau B, Guicheux J, Alliot-Licht B (2006). Culture medium modulates the behaviour of human dental pulp-derived cells: technical note. Eur Cell Mater.

[22] Rashid F, Shiba H, Mizuno N (2003). The effect of extracellular calcium ion on gene expression of bone-related proteins in human pulp cells. J Endod.

[23] Estrela C, Holland R (2003). Calcium hydroxide: study based on scientific evidences. J Appl Oral Sci.

